# EEG Analytics for Early Detection of Autism Spectrum Disorder: A data-driven approach

**DOI:** 10.1038/s41598-018-24318-x

**Published:** 2018-05-01

**Authors:** William J. Bosl, Helen Tager-Flusberg, Charles A. Nelson

**Affiliations:** 1 Boston Children’s Hospital, Boston, USA; 2 Harvard Medical School, Boston, USA; 3 University of San Francisco, San Francisco, USA; 4 Boston University, Boston, USA; 5 Harvard Graduate School of Education, Cambridge, USA

## Abstract

Autism spectrum disorder (ASD) is a complex and heterogeneous disorder, diagnosed on the basis of behavioral symptoms during the second year of life or later. Finding scalable biomarkers for early detection is challenging because of the variability in presentation of the disorder and the need for simple measurements that could be implemented routinely during well-baby checkups. EEG is a relatively easy-to-use, low cost brain measurement tool that is being increasingly explored as a potential clinical tool for monitoring atypical brain development. EEG measurements were collected from 99 infants with an older sibling diagnosed with ASD, and 89 low risk controls, beginning at 3 months of age and continuing until 36 months of age. Nonlinear features were computed from EEG signals and used as input to statistical learning methods. Prediction of the clinical diagnostic outcome of ASD or not ASD was highly accurate when using EEG measurements from as early as 3 months of age. Specificity, sensitivity and PPV were high, exceeding 95% at some ages. Prediction of ADOS calibrated severity scores for all infants in the study using only EEG data taken as early as 3 months of age was strongly correlated with the actual measured scores. This suggests that useful digital biomarkers might be extracted from EEG measurements.

## Introduction

Autism Spectrum Disorder (ASD) is defined by a heterogeneous constellation of behavioral symptoms that emerge over the first years of life. A recent Centers for Disease Control (CDC) report estimates that the prevalence of ASD in the United States is 1 in 68, a significant increase in the past decade^1^. As research on ASD continues at rapid pace, the etiology and developmental course appear to be increasingly diverse, resulting in a view of ASD with diverse cognitive, behavioral and neural trajectories and subtypes^2^.

High-risk infant siblings studies have demonstrated that the defining behavioral features of ASD emerge during the latter part of the first and second years of life^3^. Because ASD is behaviorally and not biologically defined, a formal diagnosis of ASD before three years of age remains challenging^4,5^ and children often do not receive a diagnosis until the preschool years or later^6^. Milder forms of ASD are particularly difficult to detect early, in part because a broad range of neurodevelopmental symptoms are common to several diagnoses, including ASD^7^. Even in cases of diagnostic uncertainty, reliable biomarkers that detect emerging ASD symptoms might be useful for developing appropriate early interventions^4^.

The brain develops rapidly during the first years of life and atypical neurodevelopment is likely due to a combination of genetics, biological, and environmental conditions, all compounded by adaptations that result from atypical interactions between the developing child and his or her environment^8^. An emerging view is that the behavioral symptoms that define ASD may be the end result of early brain adaptation, rather than the direct consequence of ongoing neural pathology^9^. This view suggests the possibility that the primary neural impairments that lead to ASD are transitory and thus difficult to detect after a critical developmental period^8^.

### Neural Correlates of Behavior

Because atypical brain development that leads to ASD symptoms is likely to precede atypical behavior by months or even years, a critical developmental window for early intervention may be missed if diagnosis or screening is based solely on behavioral features. This has fueled a search for early neural correlates or biological indicators that could identify ASD in the prodromal phase.

Some models of ASD are based on atypical development of neural connectivity^10–12^, with excessive local connectivity within neural assemblies and deficits in long-range connectivity between functional brain regions implicated. Recent studies suggest the involvement of somatosensory, default mode, visual, and subcortical networks^13^. Microstructural properties of the uncinate fasciculus in 6-month-old infants were found to predict behavioral response to joint attention three months later, indicating the potential importance of this tract in social development^14^. Further, microstructural organization of the splenium of the corpus callosum was associated with atypical oculomotor function in 7-month old infants who later developed ASD^15^. These studies suggest that early atypical neural function, due to atypical neural structure, may be associated with the behavioral symptoms of ASD that appear later. The challenge from a clinical perspective is how to measure atypical neural function for use in monitoring neurodevelopment.

A recent fMRI study of 59 6-month-old infants demonstrated significant differences in the brains of children who would develop a diagnosis of ASD at 24 months of age^12^. While these results are scientifically promising, two significant challenges remain before biomarker measurement methods can be developed for clinical use. First, to be viable in a primary care setting, any brain measurement method should be low cost and simple to administer in the context of a well-baby checkup. Second, ASD is a spectrum disorder that exhibits a heterogeneous set of defining behavioral characteristics. A simple binary determination of ‘autism’ or ‘not-autism’ may not be adequate for broad clinical application, such as monitoring changing risk profiles as a child develops or response to therapeutic interventions.

### EEG for Functional Brain Measurement

Our previous studies have attempted to use traditional spectral power analysis to find early biomarkers for autism^16^. While differences were found between high risk siblings and low risk controls, they were not correlated with outcome. A recent study using data from the same infants as in the present study found that reduced frontal high-alpha power at 3 months was associated with lower expressive language skills at 12 months, but did not predict ASD-specific outcomes. Notably, the association between 3-month frontal power and expressive language skills appeared to be developmentally time-locked to the 12-month time point, with no evidence of this association persisting over the second and third years of life in our sample^17^.

**Figure 1:**
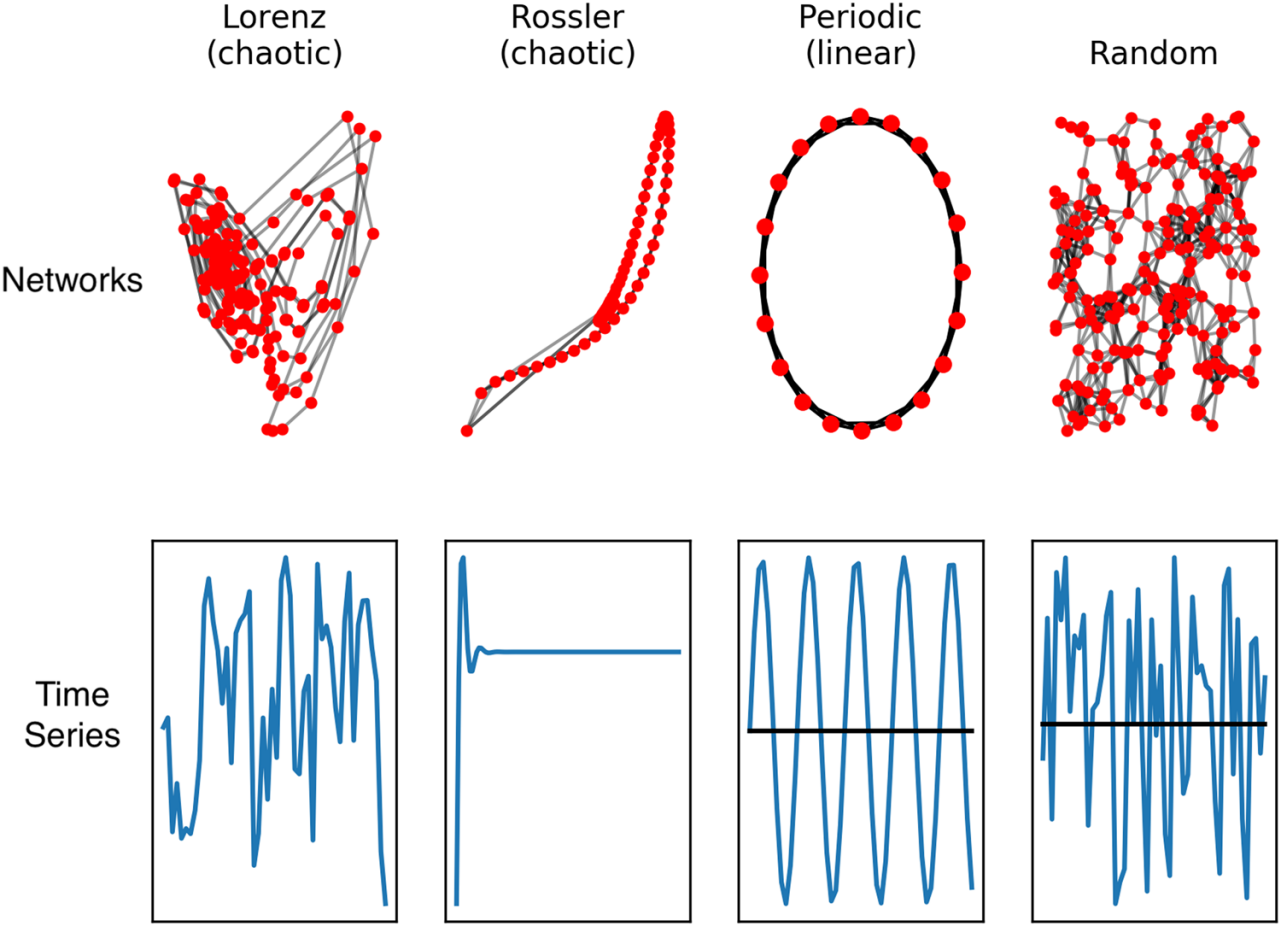
Time series and complex networks are related, and methods for reconstructing essential elements of one from the other have been developed. See, for example^18^.

The potential for EEG to be used as a functional brain imaging modality continues to improve as new methods for analyzing and extracting information from biophysical signals are developed. Methods for analyzing complex time series produced by complex networks, such as the brain, may enable network structural differences to be inferred from time series measurements^18^, as illustrated in Fig. 1. Based on this, a set of nonlinear or complex system parameters or ‘invariant measures’ from EEG signals in principle should characterize the neural dynamics of the brain that generates the signals.

The implication is that the complex time series obtained from EEG contains information about neural network structure that may provide valuable clues about atypical function^19,20^. This has clinical implications because a new generation of low cost, easy to use EEG devices are becoming available^21,22^, which would make routine brain measurements a possibility in a primary care setting.

The challenge for developmental clinical neuroscience is to determine which combinations of invariant measures and scalp locations are most relevant to the detection and monitoring of characteristics relevant to ASD. A data-driven approach may be sufficient for identifying useful clinical biomarkers. Data-driven discovery may also point in the direction of the likely neural correlates of relevant behavioral constructs or cognitive phenotypes^19^. The goal of this study is to demonstrate that nonlinear analysis of EEG signals, together with pattern classification methods, can extract information as early as 3 months of age that predicts an infant will develop a clinical diagnosis of ASD. Furthermore, the severity of ASD symptoms, as measured by the Autism Diagnostic Observation Scale (ADOS), can also be predicted from EEG data taken as early as 3 months of age, with strong correlation to the real ADOS scores that the child has at three years of age. Our results, while promising, point to many more questions about the underlying brain-behavior relationships that might explain why nonlinear EEG measures are useful digital biomarkers of ASD.

## Methods

### Participants

Participants were infants enrolled in an IRB-approved collaborative longitudinal study conducted at Boston Children’s Hospital/Harvard Medical School and Boston University. All components of the study were approved by the IRB review boards at both institutions and are covered under IRB guidelines approved by both institutions. Written, informed consent was provided by the parents or guardians prior to their child’s participation in the study.

Participants are classified into one of three outcome groups based on their original family recruitment group and the clinical determination of whether the child had a positive or negative diagnosis for ASD at the end of the study. Infants in the low risk controls (LRC) family recruitment group had at least one typically developing older sibling and no first-degree relatives with a known developmental disorder, based on a screening questionnaire. Infants recruited into the high risk for ASD (HRA) recruitment group had an older sibling with an ASD diagnosis (not due to a known genetic disorder; e.g. Fragile X syndrome). The older siblings all had expert clinical community diagnoses, which were confirmed by a member of the study staff using the Social Communication Questionnaire (SCQ)^23^ or the Autism Diagnostic Observation Schedule (ADOS-2)^24^. Once enrolled, infants were scheduled for visits from 3 to 36 months of age. Children who completed at least two of the visits (scheduled at 3, 6, 9, 12, 18, 24, 36 months) and completed one visit at 18 months or later, during which they were given a clinical evaluation for ASD, were included in this study. Most infants completed the final 36-month evaluation for ASD.

There were 3 outcome groups: LRC−: infants from the low risk recruitment group who did not receive an ASD diagnosis; HRA−: infants from the high-risk recruitment group who did not receive an ASD diagnosis; ASD: infants from either recruitment group who received an ASD diagnosis. Table 1 shows the number of participants in each of the family recruitment groups, their distribution into outcome groups, and the number of participants at each age. Note that not all participants provided data at every scheduled visit. The distribution of ages for the latest clinical diagnosis is also illustrated in Table 1.

**Table 1:** The distribution of the 188 participants from each of the family recruitment groups is shown by (a) outcomes and (b) the number of EEG measurements available for analysis from each evaluation age.

(a) Family Recruitment Group		Outcome groups		
		LRC− LRC, not autism	ASD Autism diagnosed	HRA− HRA, not autism
Low Risk Controls (LRC): 89		86	3	—
High Risk for Autism (HRA): 99		—	32	67
Age of clinical outcome determination	36 m	69	29	52
	24 m	9	3	11
	18 m	8	3	4
**(b) EEGs available and analyzed by age**		**LRC**−	**ASD**	**HRA**−
**Age of EEG**	**Number of subjects at age**			
3	41	14	11	16
6	131	69	8	43
9	154	73	26	53
12	158	77	31	49
18	125	56	25	44
24	129	60	26	43
36	138	65	31	42

### Clinical Evaluations

ASD diagnoses for the participants were based on the ADOS^24^ and expert clinical judgment at 18, 24, or 36 months, whichever is latest. The ADOS is a semi-structured, standardized assessment that consists of social and play activities to elicit behaviors related to diagnosis of ASD. In addition to a binary diagnosis of either ASD or not-ASD, a Calibrated Severity Score (CSS) with numerical range 1–10 was obtained from the ADOS for each participant. The CSS offers a method of quantifying ASD severity with relative independence from individual characteristics such as age and verbal IQ^25^.

The ADOS was administered by research staff with extensive experience in testing children with developmental disorders and co-scored by an ADOS-reliable researcher via video recording. Cases of concern (those meeting criteria on the ADOS or coming within 3 points of cutoff) were reviewed by a licensed clinical psychologist who evaluated video recordings of behavioral assessments along with the scores from those assessments to determine final clinical judgment: no clinical concern/typically developing, ASD, or non-spectrum concerns (e.g., symptoms of ADHD, anxiety, language delay). Infants were included in the ASD group if they had a CSS score of 4 or higher and received a final clinical judgment of ASD. In total, 150 participants received a final clinical evaluation at 36 months, 23 participants at 24 months, and 15 participants at 18 months.

Final diagnostic outcomes and EEG measurements from at least two visits were available from a total of 188 children and were included in this study. For the purposes of this study, all visits were treated as independent encounters. For example, all EEG measurements taken at 12-month visits were used to predict outcomes independent of measurements taken at other ages for the same participant. Although a growth trajectory analysis was beyond the scope of this study, we performed one classification test by combining measurements from 6 months and 9 months into a single set of features for subjects who had both 6- and 9-month visits.

### EEG Collection Procedure

Infants were seated on their mothers’ laps in a dimly lit, electrical- and sound-shielded testing room. An experimenter was in the room and blew bubbles throughout the procedure to maintain the infants’ interest, limit movement and increase tolerance of the electrode net. The baby’s head was measured and marked with a washable wax pencil to ensure accurate placement of the net, which was then placed over the scalp. Prior to fitting the sensor net over the scalp, the sponges were soaked in electrolyte solution (6cc KCL/liter distilled water) in order to facilitate electrical contact between the scalp and the relevant electrode. Scalp impedances were checked on-line using NetStation software (EGI, Inc, Eugene OR), the recording software package that runs this system. For most participants, at least two minutes of baseline activity were recorded, but depending on the willingness of the infant, recording periods may have been limited to two minutes.

Continuous EEG was recorded using either a 64-channel Geodesic Sensor Net or a 128-channel HydroCel Geodesic Sensor Net (Electrical Geodesics Inc., Eugene, OR; https://www.egi.com/research-division/geodesic-sensor-net) referenced online to vertex (Cz). The data were amplified, filtered (band pass 0.1-100.0 Hz), and sampled at a frequency of either 250 Hz or 500 Hz. The latter were downsampled to 250 Hz before further analysis. Signals were digitized with a 12-bit National Instruments Board (National Instruments Corp., Woburn MA). Data from 19 sensors uniformly distributed across the scalp were used in our analysis. The locations of the sensors chosen for analysis are the standard 10–20 montage: Fp1, Fp2, F7, F3, Fz, F4, F8, T7, C3, Cz, C4, T8, P7, P3, Pz, P4, P8, O1, O2. The same subset of sensors was used regardless of which Geodesic Sensor Net was used to measure the data.

### EEG Data Analysis

Each of the EEG samples was processed in an identical manner by the following steps. Continuous 30-second segments were selected from the beginning of each of the EEG recordings for each subject when the child was sitting quietly. No splicing of segments or selection based on review was performed.

### Frequency Bands

The signal or time series from each sensor was decomposed into multiple frequency bands using a wavelet transform^26^. We note that the Haar wavelet transform yields a multiscale decomposition that is mathematically equivalent to the coarse-graining procedure introduced by Costa *et al*.^27^ for multiscale entropy analysis of biological signals. The coarse-graining procedure for signal decomposition has been used in previous studies for multiscale entropy analysis of EEG^28^. In the present study, we used the Daubechies (DB4) wavelet, which is very similar to the Haar wavelet, but is more commonly used for signal analysis^29^.

Using the wavelet transform, each sensor signal is decomposed into six power-of-two frequency bands that are approximately equal to the commonly used definitions of delta, theta, alpha, beta, gamma, and gamma + bands used for EEG analysis. The specific software used for the wavelet transform is publicly available, along with instructions and python code, at: https://pywavelets.readthedocs.io. Table 2 shows the six frequency bands used in this study. For the remainder of this paper the standard frequency band labels will be used, but these refer specifically to the power-of-two bands shown in Table 2.

**Table 2:** Raw EEG signals are collected with a sampling rate of 256 samples per second, then decomposed by powers of two into the frequency bands shown using a wavelet decomposition. The resulting frequency bands are approximately equivalent to the standard EEG frequency band labels.

Wavelet level	Wavelet frequency range	Approximate EEG label
1	64–128 Hz	high gamma
2	32–64 Hz	gamma
3	16–32 Hz	beta
4	8–16 Hz	alpha
5	4–8 Hz	theta
6	0–4 Hz	delta

### Nonlinear Signal Features

Nine nonlinear features were computed for each of the frequency bands derived from each of the 19 sensors used in our analysis. A broad range of nonlinear features was computed to give as complete a characterization of the signal dynamics as possible. The features computed included:

(i) Seven values derived from Recurrence Quantitative Analysis (RQA). RQA is an empirical approach to analyzing time series data that is in principle capable of characterizing all of the essential dynamics of a complex system. It has been found to be useful for analyzing “real-world, noisy, high dimensional data”^30^. Software for computing recurrence plot statistics is publicly available in the python package pyRQA 0.1.0^31^. A more complete discussion of RQA analysis may be found in the literature^30,32^. Seven of the most commonly used recurrence plot values (RR, DET, LAM, L_max, L_entr, L_mean, and TT) were computed and used in this study.
(ii) Sample entropy and Detrended Fluctuation Analysis, denoted by SampE and DFA, respectively, were also computed. Sample entropy was the sole nonlinear feature used in our previous analysis of a subset of the data analyzed for this study^33^. DFA quantifies a different signal property and is a measure of the “long-term memory” of a time series. It can be used to determine whether the time series is more, less, or equally likely to increase if it has increased in previous steps. SampE and DFA were computed using publicly available software “*Nonlinear measures for dynamical systems”* or nolds, version 0.3.2, which can be downloaded from (https://pypi.python.org/pypi/nolds).

These 9 measures and their general meaning are summarized in Table 3. In principle, these measures characterize the dynamical features of the EEG time series, which should correlate to functional characteristics of the neural network or brain that produced the time series. Although these measures are well-established physical and mathematical values, their meaning in the context of neural science has only recently begun to be explored. To our knowledge, this study is the first application of RQA and the other measures described here to developmental neuroscience.

**Table 3:** Nonlinear invariant measures of a time series and their physical interpretation.

Nonlinear Invariant Variable	Symbol	Description
**Invariant measures computed with the NOLDS software package**		
Sample Entropy	SampE	Sample entropy measures the complexity of a time-series, based on approximate entropy as discussed in our previous study^28^
Detrended Fluctuation Analysis	DFA	The hurst exponent is a measure of the “long-term memory” of a time series. It can be used to determine whether the time series is more, less, or equally likely to increase if it has increased in previous steps. DFA measures the Hurst parameter *H*, which is very similar to the Hurst exponent. The main difference is that DFA can be used for non-stationary processes
**Invariant measures derived from Recurrence Quantitative Analysis (RQA)**		
Entropy derived from recurrence plot	L_entr	A measure of entropy derived from the diagonal lines of the recurrence plot, most closely associated with the Shannon entropy. It is related to other measures of entropy, such as the sample entropy above.
Max line length	L_max	Lmax is related to the largest Lyapunov exponent of a chaotic signal, which is a dynamic complexity measure that describes the divergence of trajectories starting at nearby initial states^69^. Higher Lyapunov exponents (lower Lmax values) are typically associated with pathological conditions^70,71^.
Mean line length	L_mean	The time that two segments of the recurrence plot trajectory are close to each other, and can be interpreted as the mean prediction time of the signal, a measure of chaos or divergence from an initial point
Recurrence rate	RR	The probability that a system state recurs in a finite time. RR has been found useful for detecting evoked response potentials (ERPs) using single trials^72^. The concept is similar to periodicity, but is more general.
Determinism	DET	DET comes from repeating patterns in the system and is an indication of its predictability. Regular, deterministic signals, such as sine waves or deterministic chaos, will have higher DET values, while uncorrelated time series, such as random numbers, will cause low DET.
Laminarity	LAM	Laminarity represents fast transitions and instabilities, or the frequency of transitions from one state to another, without describing the *length* of these transition phases. More frequent appearance of laminar states may relate to more frequent “seeds” for synchronized dynamics^73^.
Trapping time	TT	Trapping time is an estimate of the time that a system will remain in a given state, such as the length of transition states, as opposed to the time for the transition to take place (compare to LAM).

### Feature Selection and Classification

The total feature set computed from each EEG session consists of 9 nonlinear values computed on 6 scales or frequency bands for each of 19 sensors, totaling 1026 features. Many of these features are likely to be correlated. For example, spatially close sensors might have correlated signal properties. SampE and L_entr are both measures of entropy, though derived by entirely different algorithms. Discovering the most informative features for predicting emerging ASD was accomplished using feature-ranking methods.

Several different learning algorithms were initially tested and compared for this study, including k nearest neighbors, random forest, and support vector machine. All were found to give similar classification results. For the remainder of this paper, all classification and prediction results reported were computed using the support vector machine (SVM) method with radial basis functions. Feature selection was done with a recursive feature elimination algorithm as described in^34^, using the rfecv method in the Python open source package scikit-learn (www.scikit-learn.org). Scikit-learn was used for all machine learning calculations, using all default parameters.

### Prediction of ASD and Symptom Severity

**Figure 2:**
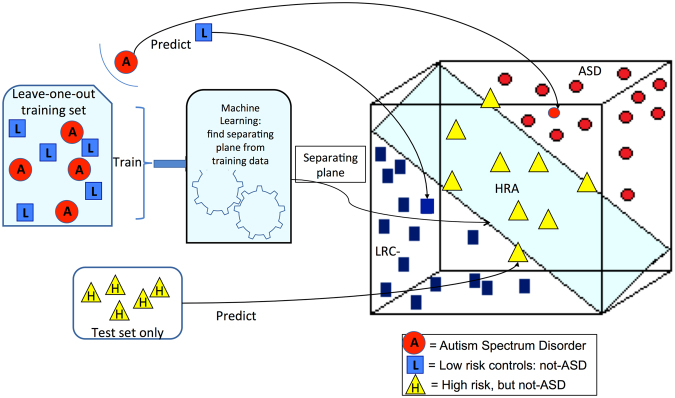
A schematic representation of classification with a support vector machine (SVM) method is shown with 3 dimensions representing 3 features. The training set is used to create a model or separating hyperplane in the feature space. Axes of the feature space are the nonlinear EEG features. Test subjects are then classified by determining which side of the plane the subject’s features places them. ASD and LRC- subjects are used for the training set. Leave-one-out cross validation leaves out a single subject from the training set and then makes a prediction for the left-out subject. The distance of a subject from the hyperplane can be used to estimate severity. This distance was scaled to approximate the Calibrated Severity Score (CSS), where 1.0 is the lowest score for children with no autism symptoms and 10 is the highest score for the most severe autism cases.

A prediction of the binary diagnostic outcome, either ASD or not-ASD, was computed from the nonlinear features described above using a leave-one-out cross validation procedure. The cross-validation procedure is illustrated in Fig. 2 using three dimensions to represent three features. The procedure is as follows.

The training set is used to find the model or separating hyperplane that divides the feature space into halves. The SVM method is a formal algorithm for finding the plane that best separates the two groups. The plane that separates two groups is illustrated in Fig. 2. We note that the axes in this illustration represent the nonlinear EEG features. In practice, there are as many dimensions as there are features. The training set of data containing data points for each outcome group is used to define the plane. New data points can then be classified using their EEG features to determine the location in feature space, which lies on one side of the plane or the other.

Ideally, the training set is a large set used to find the model plane and the test set is an entirely new set of subjects that are classified to find sensitivity and specificity of the model. When such large independent data sets are not available, cross validation is a process whereby a single subject is left out of the training set, and the resulting plane is then used to classify the left-out subject. This process is repeated for every subject. For our data, ASD and LRC− subjects are used for the training set with a leave-one-out cross validation scheme. The HRA− subjects are also classified, using all the ASD and LRC− subjects for the training set. Thus, the outcome of every subject is predicted from data derived from the other subjects of the same age group. This prediction can then be compared to the known outcome and determined to be true positive, true negative, false positive, or false negative. Sensitivity, specificity and PPV are computed from these quantities with standard definitions.

The distance of a subject from the hyperplane can be used to estimate severity. This distance was scaled to approximate the Calibrated Severity Score (CSS), where 1.0 is the lowest score for children with no autism symptoms and 10 is the highest score for the most severe autism cases. Prediction of CSS values used the same training sets and leave-one-out cross validation as was used for the classification of binary outcomes.

### Significance Testing

The significance of the classification results for each method was estimated empirically using the permutation approach described in^35^. Because classification accuracies were high, the empirical p-value in every case was 0, or p < .01 since 100 trials were used.

### Multiscale and Frequency Bands

**Figure 3:**
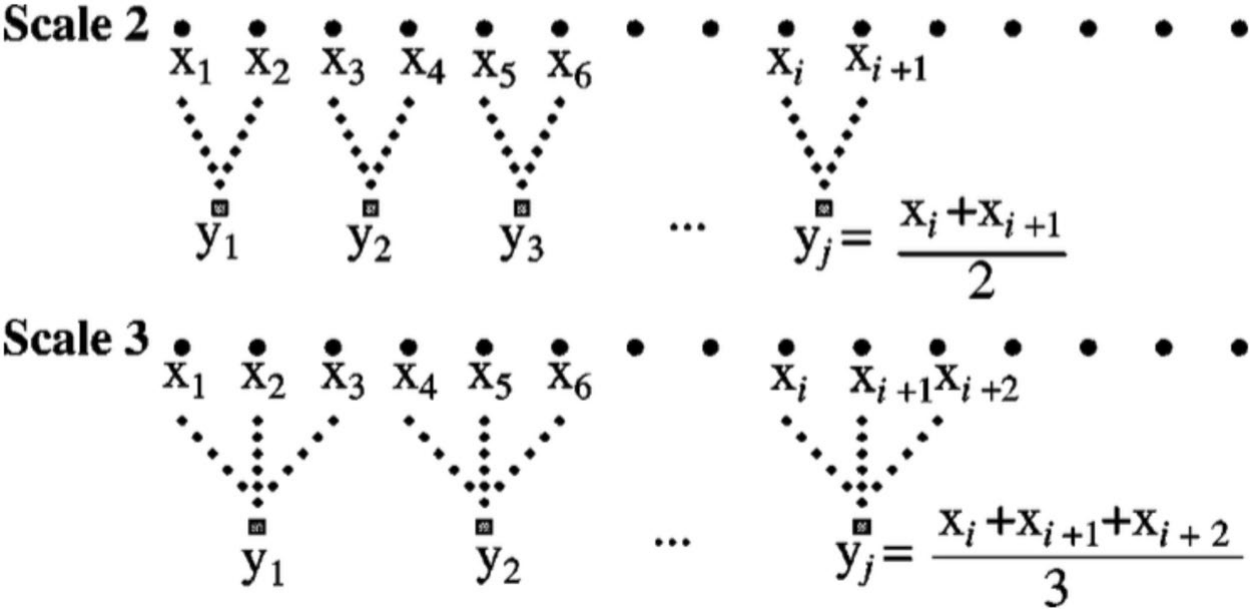
The coarse-graining procedure introduced by Costa *et al*.^27^ is illustrated. For powers of 2, the resulting scaled time series are identical to Haar wavelet transform approximations.

Multiscale entropy was first described by Costa *et al*.^27^ as a method for analyzing physiological signals. The scaling procedure generally uses the coarse-graining or averaging algorithm illustrated in Fig. 3. For power-of-two scales (2, 4, 8, 16, …) the time series produced by this procedure are identical to the Haar wavelet transform^26^. Multiscale analysis is applied to all of the measures computed in this paper, including sample entropy, which is the primary measure for multiscale entropy.

The connection between the coarse-graining algorithm and wavelets is important because a great deal is known about the properties of wavelet transforms. In particular, wavelets can be associated with common frequency bands. For example, if an EEG signal has a sampling rate of 256 Hz, then the signal contains frequencies up to 128 Hz, as determined by the standard Nyquist limit^36^. Scale 2 in the coarse-graining procedure is equivalent to wavelet approximation level 1, and contains all frequencies up to one-half of the original signal, 0 to 64 Hz. The averaging process removed the highest frequencies. Continuing the averaging process gives the results shown in Table 4, which shows the relationship between coarse-graining scales and frequency bands. The approximate frequencies used in neurophysiology, delta (0–4 Hz), theta (4–7 Hz) and so on, are also shown in Table 4. We note that the signals recorded in our study were hardware bandpass filtered in the 0.1–100 Hz range. Thus, the band labeled high gamma has signals in the 64–100 Hz range, and the delta band has signals in the 0.1–4 Hz range.

**Table 4:** The coarse-graining procedure is mathematically identical to the Haar wavelet transform. For signal collected with a sampling rate of 256 samples per second, the frequency bands contained in the scales, wavelet approximations, and wavelet details are shown.

Coarse-grain averaging scales	Wavelet level	Frequency range		
		Wavelet approximation	Wavelet detail	
			Frequencies	EEG Band
1	0	0–128 Hz	original signal	
2	1	0–64 Hz	64–128 Hz	high gamma
3	2	0–32 Hz	32–64 Hz	gamma
4	3	0–16 Hz	16–32 Hz	beta
8	4	0–8 Hz	8–16 Hz	alpha
16	5	0–4 Hz (delta)	4–8 Hz	theta

Wavelet transforms decompose a time series similarly to the coarse-graining procedure, but also retain the details that have been removed in each step. Wavelet *approximations* are equivalent to the multiscale ranges, while the wavelet *details* contain the higher frequencies that have been removed. Thus, the multiscale procedure introduced by^27^ may be identically replaced by a wavelet transform if the approximations are used. Alternatively, multiscale values might be computed on the wavelet details, which contain frequency ranges that are similar to the traditional EEG signal bands.

For this study, we used the more appropriate DB4 wavelet transform to derive the wavelet details, which are power-of-two frequency bands. The closest traditional labels (delta, theta, alpha, beta, gamma, and gamma-plus) to the wavelet detail are shown in Table 4. For the remainder of this paper, six non-overlapping frequency bands are used for all analysis and the traditional frequency band labels are used in figures. The original signal, denoted by scale 1 in the coarse-grain procedure or the level 0 wavelet, was included in our early analysis, but the feature ranking and selection algorithms never chose the full spectrum original signal for classification. Thus, only the wavelet details as shown in Table 4 are considered further.

### Required Software Packages

All computations performed for this study were carried out using python code. All required calculations used standard python packages, including numpy and scipy, and other publicly available packages listed here:

eegtools (for reading edf files): https://github.com/breuderink/eegtools

pywavelets (for wavelet decomposition): https://pywavelets.readthedocs.io

pyrqa (RQA calculations): https://pypi.python.org/pypi/PyRQA/

nolds (Sample entropy and DFA calculations): https://pypi.python.org/pypi/nolds

scikit-learn (feature ranking and machine learning): http://scikit-learn.github.io/stable

Unless noted otherwise, default parameters were used for all function calls, such as feature selection and machine learning computations.

### EEG Data Availability

The methods described above can be applied to any set of EEG data, together with diagnostic labels. The specific participant data used for our study was consented specifically for use by researchers affiliated with the Laboratories of Cognitive Neuroscience at Boston Children’s Hospital or the Tager-Flusberg laboratory at Boston University. For this reason, the raw EEG data cannot be released publicly.

## Results

Three principle results were found and are reported here.

1. *Early detection of emerging ASD*. Classification methods were able to distinguish the ASD from the LRC− infants with nearly 100% sensitivity and specificity using EEG measurements from each age beginning at 3 months. The HRA− outcome group was also classified with significantly high accuracy, but presents challenges for cases for which the EEG data places them near the diagnostic borderline. A dip in predictive accuracy is seen at 12 months of age.
2. *Quantitative Estimate of CSS*. The severity of ASD symptoms, as determined by the CSS, was predicted from EEG measurements taken from each age beginning at 3 months. The predicted scores correlated relatively strongly with the actual CSS scores.
3. *Significant Differences in ASD Features*. Significant differences were found between the ASD and LRC− groups for many of the nonlinear measures computed in this study. An apparent shift in the difference between ASD and not-ASD mean group values is evident at about 12 months of age.

These results are described in more detail in the following sections.

### Early Detection of Emerging ASD

Participants in this study were measured at 3, 6, 9, 12, 18, 24, and 36 months, but not every participant was measured at every possible age. Thus, for predictions, the set of all measurements at each age was treated independently and used in a leave-one-out cross validation computation to determine predictive accuracy of the features. Three sets of classification results are shown in Table 5. As noted previously, a classification experiment was also performed by simply combining 6- and 9-month measurements for subjects that came for both of these visits.

**Table 5:** Predictive classification of 36-month outcomes based on EEG feature classification. Sens = Sensitivity, Spec = Specificity, PPV = Positive Predictive Value. The age represents the age at which EEG was taken. The ‘6 + 9’ row was derived by combining all nonlinear features from measurements at 6 and 9 months from participants who had both visits. Columns (a) are results from classifying low risk controls without autism (LRC−) and ASD infants. (b) results from classifying the outcome of all participants. The last set of results, (c) was obtained by computing simulated CSS scores based on distance from the model hyperplane, as illustrated in Fig. 2, then removing participant scores that fell in the 3.5 to 4.5 range. These were labeled as “uncertain”. Sensitivity, specificity, and PPV were then computed for all of the remaining participants.

Age of testing	N subjects			(a) ASD & LRC− only			(b) ASD & (LRC− + HRA−)			(c) ASD & (LRC− + HRA−) with uncertain HRC− removed			Number of Uncertain HRA−
	LRC−	ASD	HRA−	Sens	Spec	PPV	Sens	Spec	PPV	Sens	Spec	PPV	
3	14	11	16	0.82	1.0	1.0	0.82	0.99	0.97	0.82	0.92	0.82	4
6	70	18	43	1.0	1.0	1.0	1.0	0.99	0.95	1.0	0.99	0.95	15
9	75	26	53	0.96	1.0	1.0	0.96	0.90	0.65	0.96	1.0	1.0	28
6 + 9	62	15	39	1.0	1.0	1.0	1.0	0.96	0.79	1.0	1.0	1.0	7
12	78	31	49	0.87	1.0	1.0	0.87	0.90	0.71	0.90	0.93	0.78	17
18	56	25	44	1.0	1.0	1.0	1.0	0.90	0.71	1.0	0.98	0.93	17
24	60	26	43	0.96	1.0	1.0	0.96	0.88	0.67	1.0	0.96	0.87	11
36	65	21	42	0.95	1.0	1.0	0.95	0.89	0.63	0.95	1.0	1.0	9

Leave-one-out cross validation was used to predict the outcome of every subject from data derived from the other subjects of the same age group. This prediction can then be compared to the known outcome and determined to be true positive, true negative, false positive, or false negative. Sensitivity, specificity, and positive predictive values were computed from the usual definitions.

Set (a), labeled “ASD and LRC− Only”, used only these two outcome groups in the classification calculations. These two groups were considered the end-point cases with either no ASD symptoms, or a confirmed diagnosis of ASD. At every age from 3 to 36 months, classification accuracy between LRC− and ASD groups was excellent, with perfect specificity and positive predictive value (PPV). However, an apparent dip in sensitivity occurs at 12 months. For subjects that participated in both 6- and 9-month visits, EEG measurements were also combined into a single set containing nonlinear features from both measurements as a simple test of how accuracy might improve with measurements from more than one age.

The calculations for the next classification, labeled (b) ASD & (LRC− + HRA−) in Table 5, included all participants. The final predicted binary outcome of the HRA− participants is significantly accurate, but less so than for the two other outcome groups (LRC− and ASD), resulting in lower overall specificity and PPV. The accuracy of the HRA− outcome predictions was better at younger ages (3 to 9 months), then dipped in accuracy starting at 12 months.

As will be explained more fully in the next section, the distance of a participant’s EEG features from the separating plane can be computed to provide a simulated CSS score. In general, a score of 4 or above is considered indicative of ASD. Using this criterion, a score between 3.5 and 4.5 might be considered “too close to call” or uncertain. Using this threshold, scores in this range we labeled as ‘uncertain’ and the remaining participants were classified. The results of this classification are in the columns of Table 5 denoted by (c). For these results, every participant was labeled as either ASD, not-ASD, or uncertain. We note that when 6 and 9 month features for each participant were combined, the classification accuracy was 100%, with only 7 of 39 HRA− infants labeled uncertain. Predictions at 12 months of age were consistently the lowest.

In summary, the classification of the two primary groups, ASD and LRC− was high at every age from 3 months to 36 months. HRA− infants were more difficult to classify, perhaps because some exhibit subtle ASD symptoms and thus their EEG features are intermediate between not-ASD and ASD features (Charman *et al*., 2016). Empirical p-values^35^ were exactly 0.0 after 100 trial classifications with shuffled labels for every method at every age, demonstrating that the classification accuracy was unlikely to be due to chance.

### Quantitative Estimate of ADOS CSS

While an early binary prediction of a future diagnosis of ASD is of clinical value, an early estimate of future severity of ASD symptoms might be of greater utility for planning services and interventions, as well as for monitoring changes related to early therapy.

**Figure 4:**
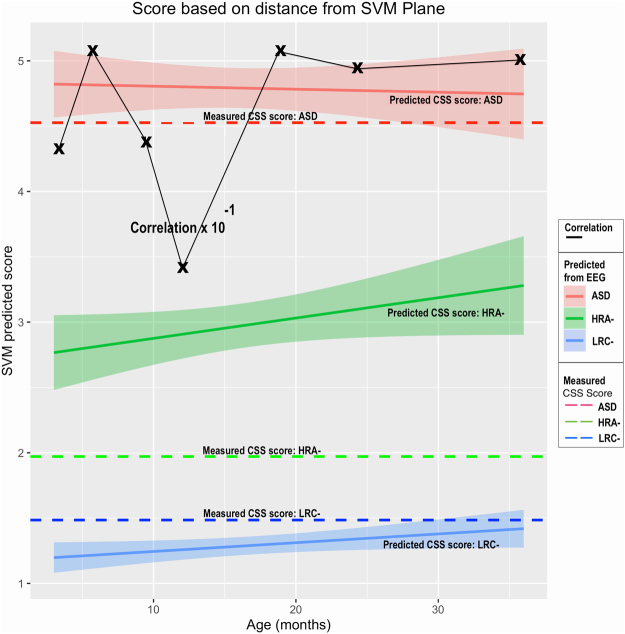
Measured CSS scores for each outcome group (solid lines), along with predicted scores derived from EEG features at each age are shown (dashed lines). Confidence intervals are shaded for predicted values. The age refers to the age at which EEG data was collected and used for the prediction. Correlation coefficients between predicted scores and the measured CSS values are shown by Xs if the axis values are multiplied by 10^−1^.

Using the method described previously, and illustrated in Fig. 2, estimates of CSS values were computed from EEG nonlinear features, treating data at each age independently as before. A plot of predicted CSS values, along with confidence intervals, are shown in Fig. 4. The measured CSS values (with assessment ages as noted in the methods section) are also shown as solid colored lines for reference. Correlation coefficients were computed for measured CSS values for each child and the predicted values. These values are shown as Xs connected by a black line.

Predicted LRC− CSS scores are quite low, with narrow confidence intervals, as are the assessed CSS values. Similarly, the predicted ASD scores average close to 5, similar to the actual CSS values. Predicted scores computed at all ages for both LRC− and ASD at are close to the actual measured scores.

The predicted HRA− scores are intermediate between the LRC− and ASD scores, and considerably higher than the actual CSS values for the HRA− group. Correlation coefficients are strong for predictions at all ages, though we note, as in the previous analysis, a decline at age 12.

In summary, the distance from the model classification hyperplane enabled an estimated ADOS calibrated severity score to be computed from EEG features alone as early as 3 months of age. The estimated scores were strongly correlated with the actual 36-month CSS values.

### Significant Group Differences

**Figure 5:**
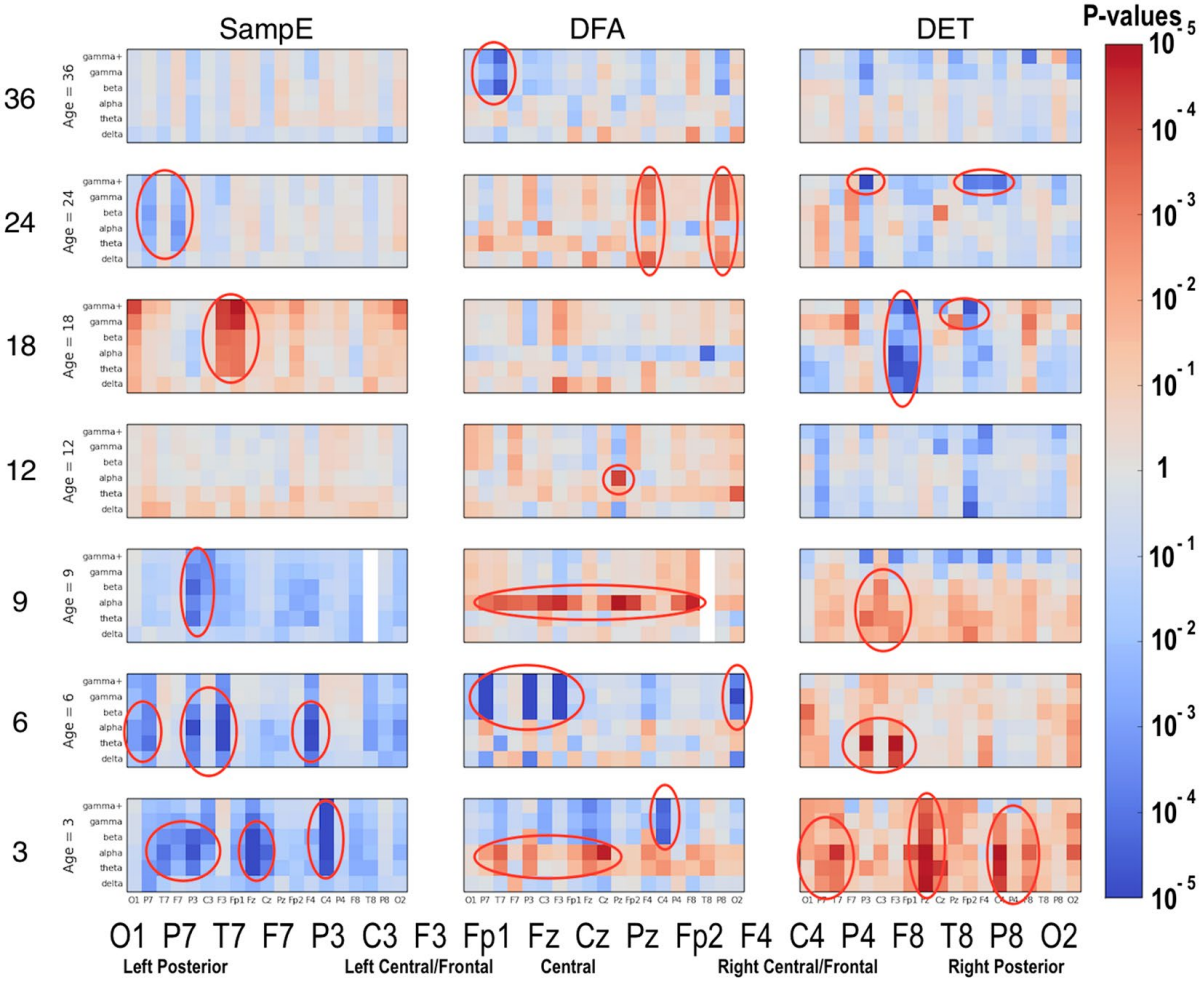
Differences between ASD and LRC− group values for SampE, DFA, and DET are shown using color for every nonlinear value. The vertical axis for each subplot represents six frequency bands, from low (delta) to high (gamma+), as defined in Table 2. The horizontal axis for each subplot is the scalp location. The axis labels are shown in a single large horizontal label across the bottom of the plot. The left or right side of each subplot corresponds to left or right sensors, respectively. Centrally located sensor values are in the center of the subplots. Red colors indicate that the values for this feature are higher in the ASD group. Blue colors indicate that ASD values are lower for that feature. Color saturation (darker red or darker blue) is correlated to the significance of the group differences. The scale on the right of the figure shows p-values of the color saturation. White areas with washed-out color indicate p-values close to 1.0, hence no significant differences.

**Figure 6:**
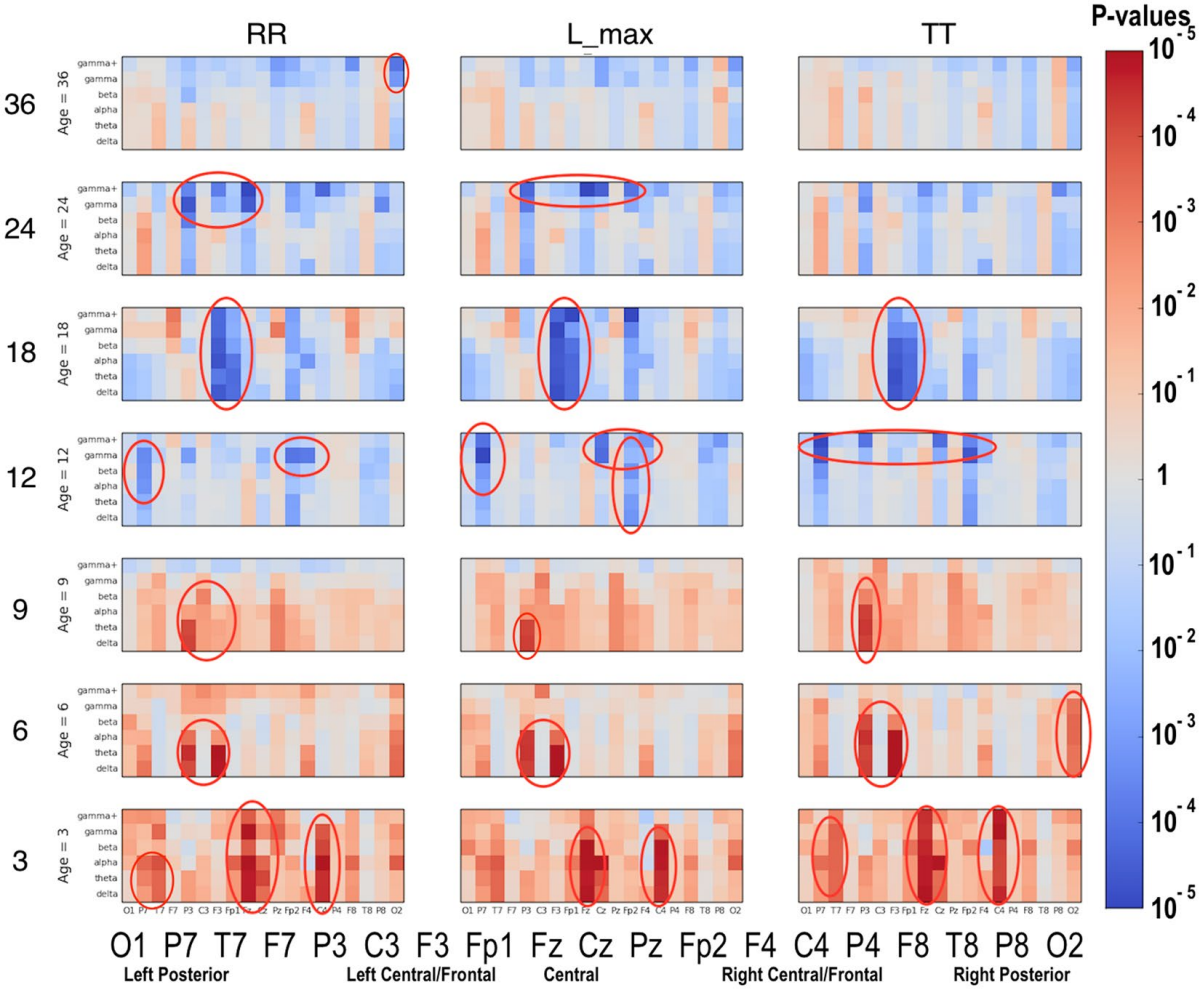
Similar to Fig. 5: differences between ASD and LRC− group values for RR, L_max, and TT are shown using color for every nonlinear value.

**Figure 7:**
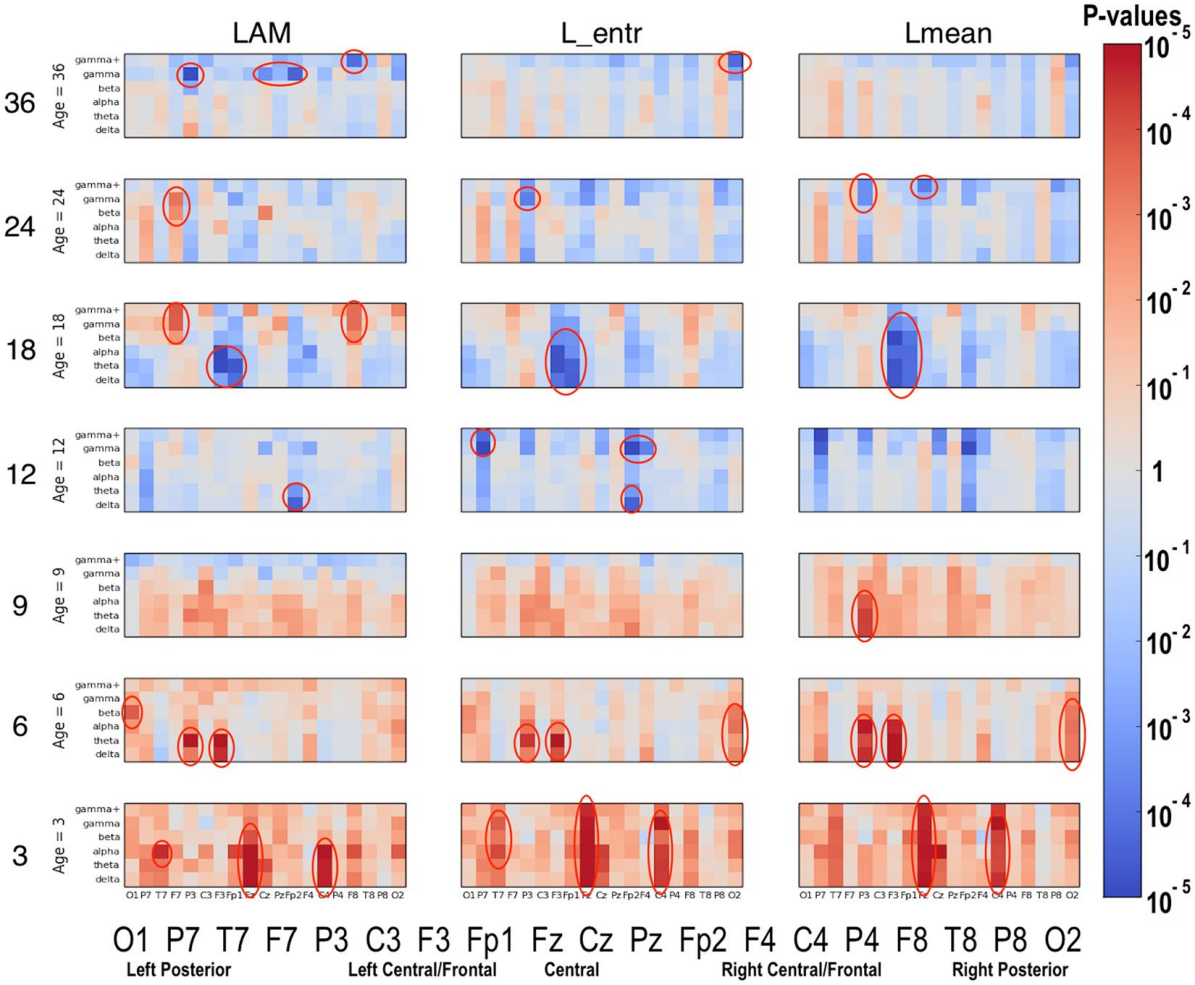
Similar to Fig. 5: differences between ASD and LRC− group values for LAM, L_mean, and L_entr are shown using color for every nonlinear value.

Significant differences between the ASD and LRC− groups were found in several sensors, frequency bands, and nonlinear measures, as shown in Figs 5–7. These plots display differences between group values using color for every nonlinear value, at every sensor location (horizontal axis of subplots) and every frequency band (vertical axis on subplots). Red colors indicate that the values for this feature are higher in the ASD group. Blue colors indicate that ASD values are lower for that feature. Color saturation (darker red or darker blue) is correlated to the significance of the group differences. The scale on the right of the figure shows p-values of the color saturation. White areas with washed-out color indicate p-values close to 1.0, hence no significant differences.

The plots can be interpreted as follows: The vertical axis for each subplot represents six frequency bands, from low (delta) to high (gamma+), as defined in Table 4. The horizontal axis for each subplot is the scalp location. The axis labels are shown in a single large horizontal label across the bottom of the plot. The left or right side of each subplot corresponds to left or right sensors, respectively. Centrally located sensor values are in the center of the subplots.

Though these plots contain a lot of detailed information, two main points are evident. First, there are many significant differences between ASD and LRC− groups, where significance level is determined by a strict Bonferroni criterion of p < 5 × 10^−5^, with 1026 features assumed to be independent. This is shown by the dark or saturated red and blue values and circled. Secondly, there is a clear shift from mostly blue to red (SampE and DFA) or from mostly red to blue (all other nonlinear values) as age increases from the 3–12 month range to 12–36 month range. That is, a shift is occurring at around 12 months of age.

### Developmental Trends in Selected Features

Several scientific questions that emerge from this work concern the fundamental neurophysiological explanations for the nonlinear features, sensors, and frequencies that are correlated with or predictive of ASD. Detailed answers are beyond the scope of this research. It is hoped that this study will encourage other researchers to contribute to this approach to help further develop the neurodevelopmental applications of nonlinear EEG analysis.

**Figure 8:**
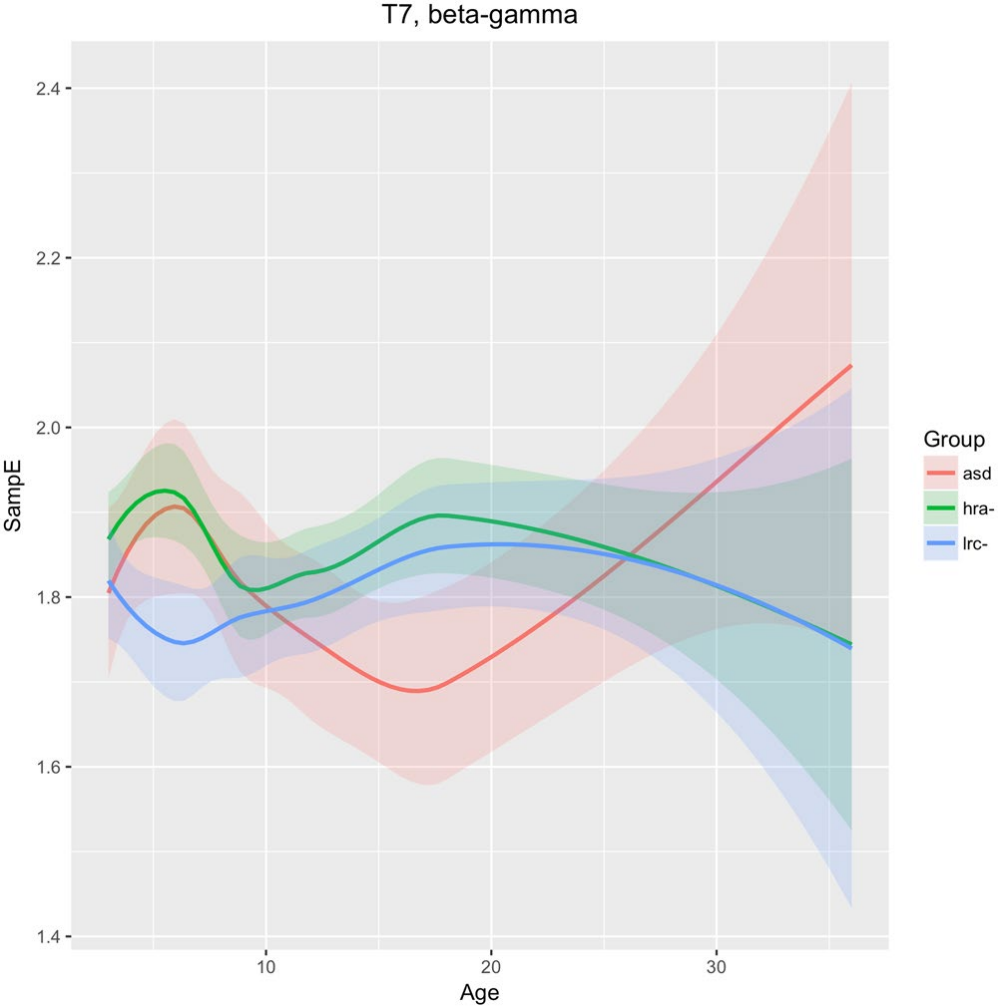
Developmental trajectories for SampE in the left temporal region (T7 sensor) in higher frequencies (beta + gamma) for ASD, LRC−, and HRA−.

**Figure 9:**
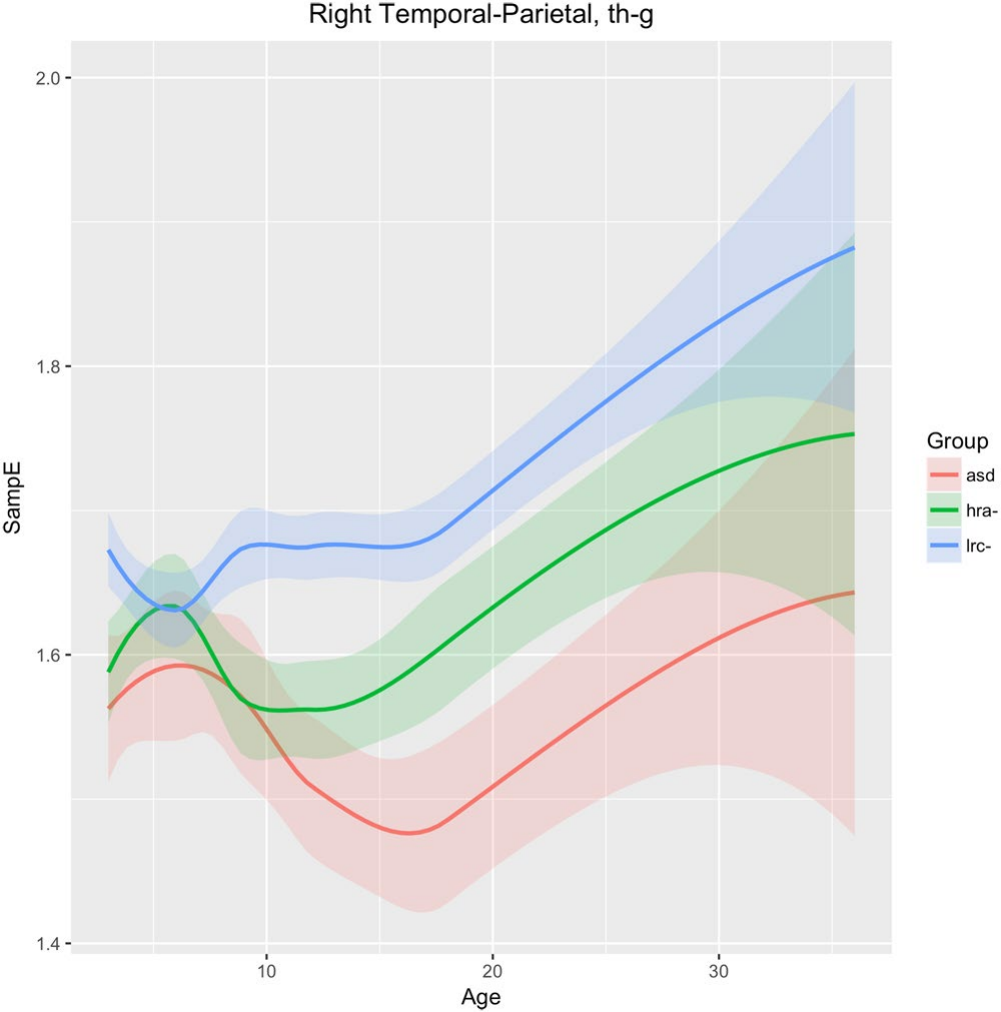
Developmental trajectories for SampE in the right temporal-parietal region (T8 + P4 + P8 sensors) in frequencies theta through gamma for ASD, LRC−, and HRA−.

**Figure 10:**
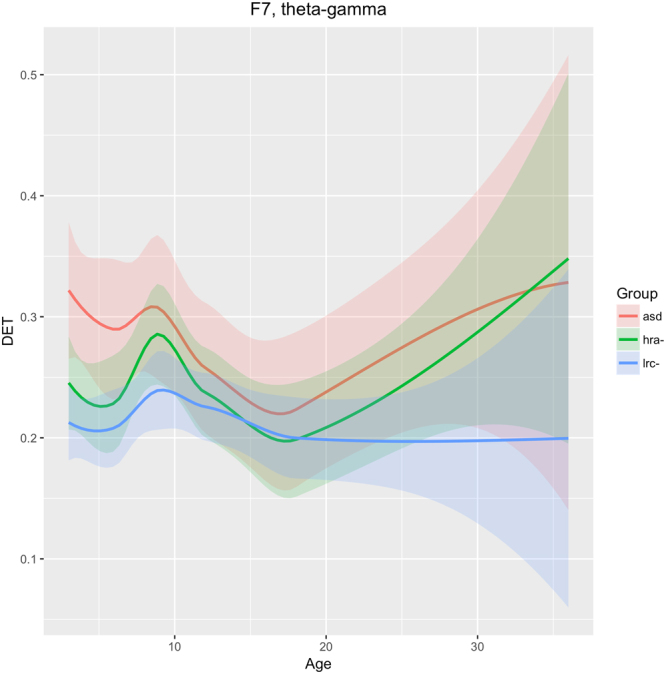
Developmental trajectories for DET in the left lateral-frontal region (F7 sensor) in frequencies theta through gamma for ASD, LRC−, and HRA−.

**Figure 11:**
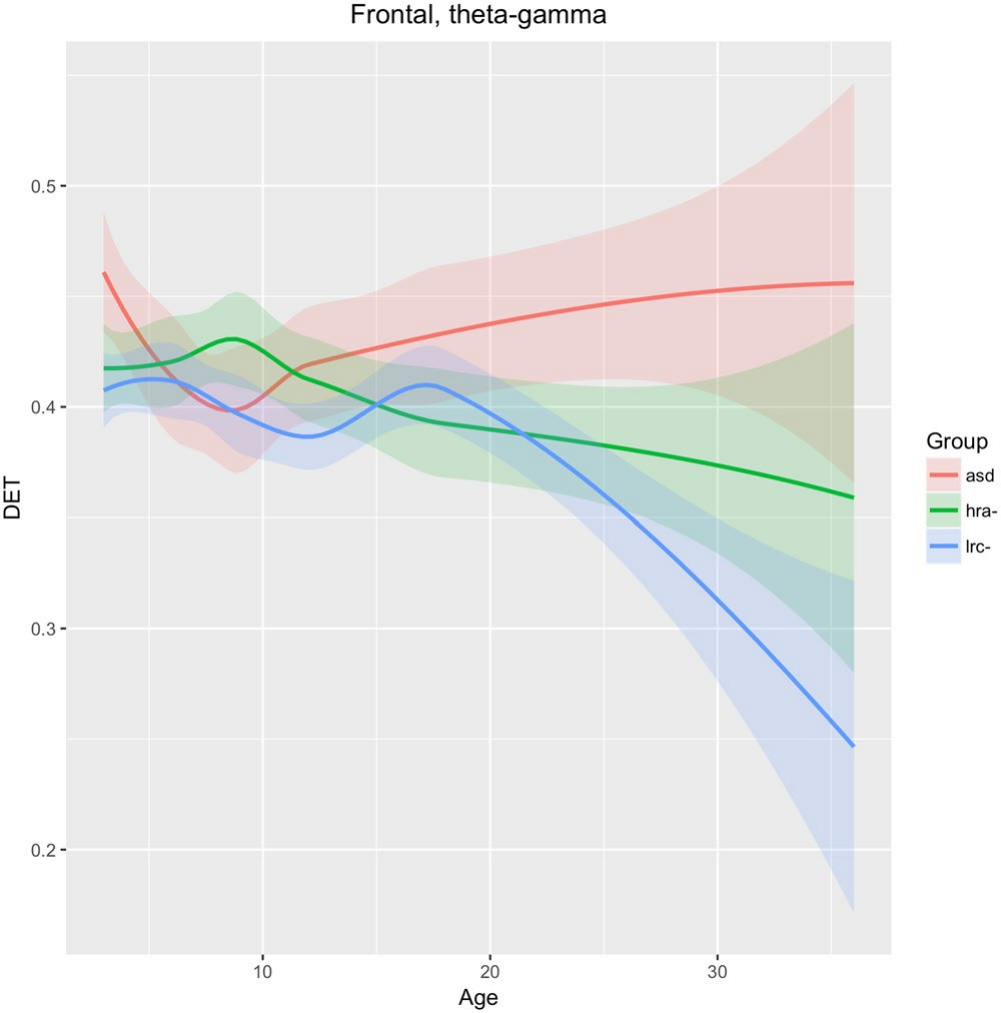
Developmental trajectories for DET in the entire frontal region (Fp1 + F7 + Fz + F8 + Fp2 sensors) in higher frequencies (beta + gamma) for ASD, LRC−, and HRA−.

**Figure 12:**
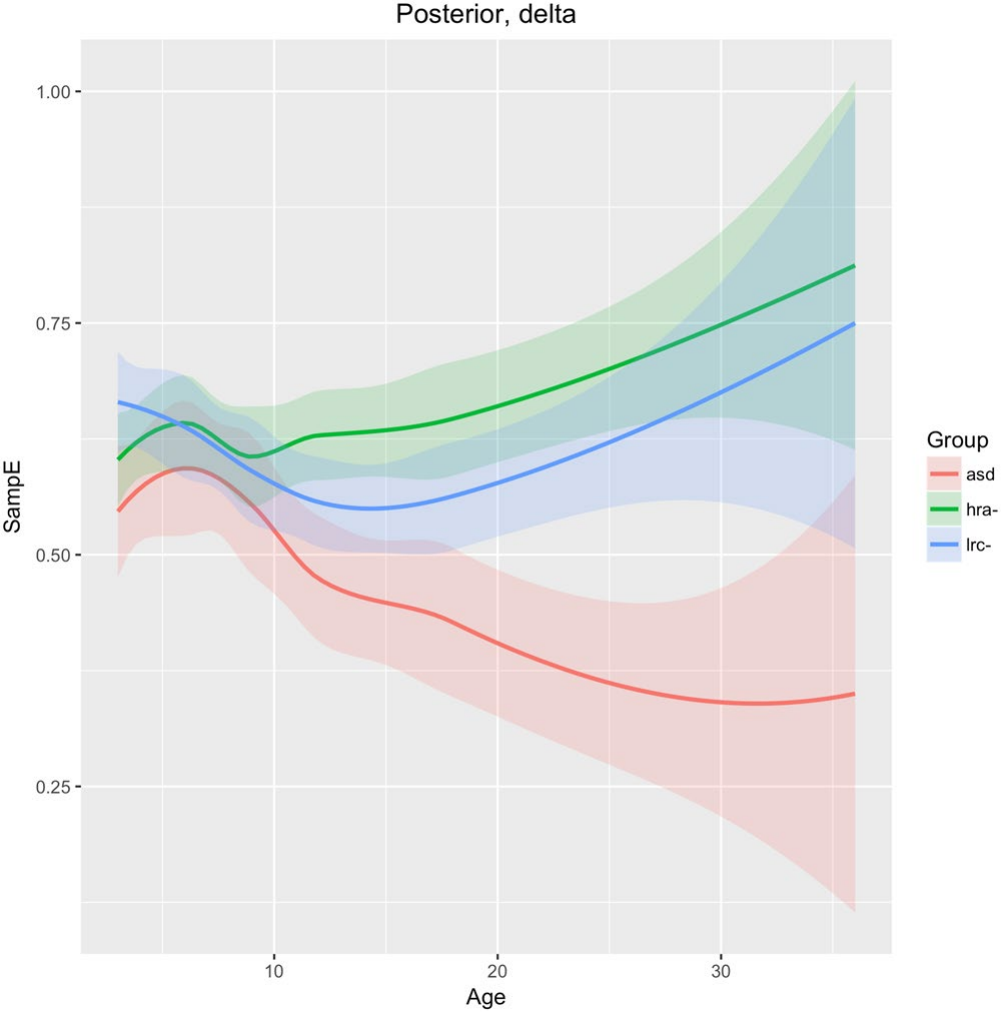
Developmental trajectories of SampE for the posterior region (O1 + O2 sensors) in low frequency (delta) for ASD, LRC−, and HRA−.

Visual inspection of Figs 5–7 reveals that several nonlinear values and sensor regions appear to exhibit significant differences among the three groups. First, we note that several features derived from RQA showed similar developmental trends (DET, L_max, RR) and thus may contain redundant information. DET may be representative of these values. Other features showed trends that did not appear to differentiate the three groups. Sample entropy appeared to follow a different trend than the RQA features and thus may represent independent information from the other measures. Thus, as an initial examination of developmental trends in these nonlinear features, SampE and DET were selected for closer review. Trends in these values in selected brain regions are shown in growth plots in Figs 8–12 and discussed briefly.

Research on physiological signals has consistently revealed that lower entropy over many scales or frequency bands is often associated with pathological conditions^27,37–39^. Higher determinism (DET) in EEG signals was found to be associated with absence epilepsy and autism^20^. Sample Entropy and DET represent different signal qualities that are not fully understood in the context of neurophysiology, but, as discussed above, appear to be independent of each other. We emphasize again that the this is only an initial evaluation of the many features that have been used in this study and further research will be required to fully explore the neurophysiological meaning of nonlinear EEG analysis.

Developmental trends in either SampE or DET are now reviewed in one of five brain regions that appear to be relevant to ASD. The plot lines shown in Figs 8–12 are derived from the value of the nonlinear feature, either SampE or DET, by averaging all the values at the sensor(s) indicated, and averaging over all the indicated frequencies. Plots for each sensor (19 of them), every frequency band (6), and every nonlinear value (9) may be revealing in future studies, but is well beyond the scope of this paper.

### Left temporal

The T7 sensor reveals that SampE in higher frequencies (beta and gamma) follows a different trajectory in the LRC− group than in the other groups. From 3–9 months, HRA− and ASD groups are similar, and different from the LRC− group. These are seen in Fig. 8. Thereafter, from 9 to 36 months, HRA− and LRC− are very close, while the ASD group first decreases from 9 to 18 months, then increases and crosses the other two trajectories.

### Right temporal-parietal

Figure 9 shows changes in sample entropy in the right temporal-parietal region (sensors T8, P4, P8). Frequencies in the theta, alpha, beta, and gamma bands were found to have similar trends and magnitudes, so are considered as a whole here. The sample entropy is similar in all three groups until 6 months, but then diverges significantly by 9 months and the groups continue to diverge through 36 months, maintaining consistent and significant differences. The ASD and LRC− groups are the farthest apart, with the HRA− group having values intermediate between the higher LRC− and the lower ASD entropy values. We note that the HRA− group appears to follow the ASD trend from 9 to 12 months, but then stops the downward trend of the ASD group and tends to normalize back toward the LRC− group, similar to the trajectory in T7 as seen in Fig. 8.

### Left frontal-temporal (F7)

We see in Fig. 10 that DET in frequencies theta through gamma is consistently lower in the LRC− group, distinct from both the HRA− and ASD groups, as early as 3 months. The trajectories of the three groups converge at 12–18 months, then diverge again. The intermediate HRA− group appears to follow the ASD group more closely than the LRC− group. These findings suggest that classification at early ages (3–9 months) would be better using features from this region, with less discriminatory ability around 12 months of age.

### Frontal

As shown in Fig. 11, DET in the entire frontal region (F7, F3, Fp1, Fp2, F4, F8) across frequency bands theta-alpha-beta-gamma are not significantly different until after 15–18 months of age, after which the values for the LRC− group drop significantly away from the other two groups. Lower DET is generally associated with healthy neural signals^20^. Values for the ASD group continues to rise moderately from 9 to 36 months. The HRA− group remains intermediate between the LRC− and ASD groups consistently after about 12 months. This seems to be consistent with the later development of the frontal region, where differentiation between groups is not apparent until after 12 months.

### Posterior region

SampE of the delta frequency band in the posterior region (O1, O2 sensors) is similar across all three groups until 9 months of age, when the ASD group diverges from the LRC− and HRA− groups, which follow similar trajectories. The sample entropy is lower in the ASD group throughout. Integrated EEG and eye-tracking studies found atypicalities in occipital delta rhythms in children with ASD^40^. The authors suggest that development in this brain region is associated with joint attention. Joint attention begins to emerge in typical infants at about 9 months and is fully developed by 18 months^41^. The consistently lower sample entropy seen in Fig. 12 may be related to underdevelopment of joint gaze in infants later diagnosed with ASD.

## Discussion

The goal of this study was to demonstrate that nonlinear values computed from relatively short segments of resting state EEG signals contain information that may be used as biomarker profiles to enable an early prediction of a future outcome of ASD. The finding that several measures of signal complexity, taken from multiple brain regions, rather than a single variable or biological parameter, is consistent with the view of ASD as an “an emergent disorder that is characterized by the loss of social communication skills in the period between 9 and 24 months … defined on the basis of alterations in the developmental trajectories across multiple domains”^42^.

Behavioral markers of ASD, such as differences in social engagement, have not yet been identified within the first year of life. It has been proposed that ASD emerges only after a typical developmental trajectory in the first 12 months becomes atypical in later infancy and toddlerhood^43^. Moreover, “these core developmental constructs appear to be more sensitive to risk status (i.e. high risk versus low risk) rather than ASD outcome”^43^. Alternatively, it may be that atypical behaviors associated with core ASD symptoms are not observed in early infancy. In either case, it is possible that subtle behavioral characteristics might introduce artifacts into the EEG signals, particularly in frontal regions due to facial muscles or eye movements. However, since efforts to explicitly measure these in young infants have so far failed, it is unlikely that artifacts could completely account for the results presented here.

A recent study using data from the same infants used in the present manuscript found that reduced frontal high-alpha power at 3 months was associated with reduced expressive language skills at 12 months, but did not predict ASD-specific outcomes. No evidence that this association persists over the second and third years of life was found^17^. An earlier study with this data examined the relationship of spectral power to outcome^16^. While group differences were found, they were not predictive of outcome. A review of EEG analysis methods for detecting ASD risk concluded that “current EEG signal analysis is not able to identify children with ASD with sufficient sensitivity or specificity to be clinically useful at this time”^44^. The methods reviewed included spectral power analysis (21 studies), functional connectivity by correlation or synchronization (12 studies), and information dynamics, which includes nonlinear methods (7 studies). Nonlinear methods were the least studied EEG analysis method, yet showed promise as an early biomarker for ASD^33,37^, and many later studies. A review of EEG methods for mild traumatic brain injury (mTBI) detection also found that spectral power measures were not adequate as a biomarker, yet nonlinear methods were described as having as yet “unknown, but high perceived potential”^45^.

The motivation for selecting the signal features was simply to use a broad range of features that might be used for functional characterization of a complex system through time series analysis^36,46,47^. Power in each frequency band was computed, but feature-ranking algorithms did not select power as a contributor to the predictions, so it was not included here. The list of features used here is unlikely to be complete, as new approaches will continue to be developed. However, multiscale analysis of entropy, DFA, and the several recurrence quantitative analysis (RQA) values are a reasonably complete list at this time. RQA is a relatively new nonlinear analysis approach that purports to give a complete characterization of nonlinear dynamical systems and might be sufficient alone^32,48^. This remains to be evaluated. RQA contains measures of entropy, a measure related to the Lyapunov exponent, and several others. In future research, we will work to optimize this list and identify the most diagnostically useful measures, as well as the most useful sensor locations and scales or frequency bands. But this will be a very large optimization problem that will likely require much larger datasets and a concerted effort by the cognitive neuroscience community together with data scientists.

It is not known how many EEG sensors, or what placement, is optimal for an effective biomarker profile to detect ASD. The 64 or 128 sensor nets chosen for this study were found to be relatively easy to place on young infants, and were well-tolerated by participants. For this study, a 19-sensor subset was initially used. From a purely computational perspective, even using 19 sensors results in a large feature set, as discussed previously and illustrated in Figs 5–7. Some studies have suggested that 19 sensors might be sufficient to detect ASD^20,49^. Thus, nineteen sensors uniformly distributed across the scalp using the standard 10–20 montage were chosen in this study as the minimum necessary to sample most of the scalp. Because predictive results were excellent with this set of sensors, there was no need to incorporate more than our initially chosen 19 sensors. Future studies may explore whether fewer sensors are adequate, or whether incorporating more sensors, perhaps focused in certain regions, would enable greater accuracy, or detection of a variety of ASD subtypes. A practical protocol for incorporating EEG measurements into primary care will have to consider issues such as cost, ease of use, and tolerance by infants and children of all ages, but these questions are beyond the scope of this study.

Although the number of infants available for the 3 month assessments is approximately one-third the number at other age groups (41 total: 11 ASD, 14 LRC−, 16 HRA), the size of the data set is still sufficient to draw meaningful conclusions. A recent study used 3-month data for analysis and found significant results^17^. The results found at 3 months are consistent with those at 6 months, and the trend from 3–6–9 and so on, is consistent. This at least suggests that significant and potentially useful information can be found at 3 months of age. Taken together, the 3-month data does not detract from results and conclusions from the other age groups, and adds support to the hypothesis that neural correlates of a later diagnosis of ASD may be found before 6 months of age, possibly 3 months or even earlier.

Although the EEG measurements used in this study are different from the functional MRI measures used in the recent study by^12^, the machine learning methodology used to predict ASD outcomes in the first year of life is similar in both studies, and relatively standard. Our approach to analyzing EEG as a set of time series produced by a complex system yields similar predictive results. Moreover, our results suggest that measurable brain activity as early as 3 months of age may be sufficient to predict the severity of ASD symptoms years later.

Recently, performance-based measures such as atypical eye gaze patterns^50–52^, unusual vocalization^51^, or fixation on geometric patterns^53^ have highlighted the many potential early markers of ASD risk. Many brain recording (ERP) and imaging techniques such as DTI^54,55^ and sleep fMRI^53^ have shown great promise to identify brain correlates of prodromal ASD, but may not be practical for routine testing of normal infants due to their cost and difficulty. The significant EEG feature differences found in our study may be neural correlates of these behavioral measures and is an area for future exploration. For example, the quantitative results in Figs 5–7 might be correlated with specific behavioral measures.

Significant differences were found in a number of EEG features, as shown graphically in Figs 5–7. Visual inspection reveals that a subtle but significant change occurs at about 12 months. More focused examination of changes in specific regions in sample entropy and determinism reveal that the convergence in the three months before and after 12 months is consistent across most regions, features, and frequency ranges. This can also be seen in the regional trajectories in Figs 8–12.

The neural interpretation of nonlinear electrophysiological results presented here require further extensive research, analogous perhaps to the micro- and macro- neural structural interpretation of Diffusion Tensor Imaging (DTI) indices that are still subject to ongoing research^56^. Nevertheless, some general interpretations are suggested by our results. Atypical development in the frontal and temporal lobes are believed to be involved in ASD^56,57^. The left frontal (Fig. 10) and total bilateral frontal (Fig. 11) trajectories appear to support this. Divergence of ASD in both of these regions appears after 18 months of age. Since frontal regions develop later than posterior regions, these may not be as useful for early detection of ASD.

Atypical lateralization of language areas has been found to be common in people with ASD^58,59^, including reduced structural asymmetry in fronto-temporal language regions, with more atypical asymmetries linked to more substantive language impairment^60^. Typical Sylvian Fissure (SF) asymmetry has been associated with typical anterior and posterior parietal asymmetry^59^. Finch *et al*.^61^ found differences in lateralization patterns of ERPs to speech stimuli across the same groups as the present study by 12 months of age, with the ASD group showing reversed lateralization compared to the LRC− group^61^. Taken together, significant differences between ASD and non-ASD subjects may be present as early as 12 months or before in temporal, frontal and temporal-parietal regions.

The differing trajectories in left temporal (T7) region for our three groups, as shown in Fig. 8, may reflect differences in a critical auditory process that develops during this time. We speculate that the deviation of the ASD development in this region from the LRC− group is related to auditory processing.

Figure 9 shows the HRA− and ASD groups following parallel paths until 9 months, significantly different from the LRC− group, in the right temporal-parietal region. Starting at 12 months, the HRA− curve breaks from the ASD trajectory and begins to parallel the LRC− group, though at an intermediate level. Both EEG and MEG studies show atypical brain activity in children with autism in primary and association auditory cortices^62^. This atypical activity may be reflected by the significantly different entropy levels found in the right temporal-parietal region after 6 months.

An apparent shift in the EEG-derived values at 12 months can be seen in the overall feature maps of Figs 5–7 as well as in some of the regional curves shown in Figs 8–12. This 12-month shift may correspond to the drop accuracy of HRA− classification as that occurs around this age. In Table 5(b), HRA− prediction is >95% for 3 and 6 month infants, then drops to between 63% and 71% thereafter (79% if 6 and 9 month values are combined). Also, the numbers of uncertain participants that are too close to the dividing plane increases considerably in the 9 to 12 month range and remains high thereafter.

One of the future challenges with our analysis is to create developmental high-dimensional trajectories that cannot be visualized in three dimensions. The trajectories in Figs 8–12 show distinct changes and differences between the three groups that may be indicators of atypical developmental paths in those regions that are associated with the later emergence of autistic characteristics.

### Predicted Severity Scores

The relatively strong correlation between predicted and actual measured CSS scores suggests that the EEG analysis presented herein may be useful not only as a means of predicting a future diagnosis of ASD, but also for assessing the severity of future symptoms. We note that a number of participants labeled as ASD had ADOS summary scores of 1 or 2, which and some non-ASD participants in the HRA− group had ADOS scores of 3 to 5. The correlations are reduced considerably by these seemingly inconsistent or heterogeneous summary scores. The predicted summary scores for the HRA− group are somewhat higher than the actual scores, which are only slightly higher than the LRC− group. It is not known which scores might be more closely indicative of actual real-world behaviors. Importantly, the predicted summary scores predict the actual group membership quite well, with relatively narrow confidence intervals around the mean.

Research suggests that features of ASD are not restricted to individuals who are diagnosed with ASD, and that there is pronounced variation within the general population relating to ASD traits, which reflect similar (though less severe) social-cognitive and behavioral features to those observed in ASDs^63^. Cognitive tests in another study revealed similarities between children with ASD and non-ASD siblings of children with ASD on standard intelligence tests, suggesting that the common cognitive profile could be an intermediate phenotype of this syndrome^64^. Studies of older children have found that siblings and parents are more likely to show mild impairments in language^65,66^, non-verbal communication (Ruser *et al*.^66^), theory of mind^67,68^ and face processing compared to controls^68^. These findings about the heterogeneity of ASD in the general population suggest that our results regarding the CSS scores might be expanded to include more refined subtypes of ASD.

The results in Table 5 warrant further examination. When training a classifier to recognize EEG features that predict a specific outcome, the training sets must be distinct. Because ASD occurs along a spectrum, training a classifier to make a binary decision (ASD or not) with subjects that have essentially the full range of ASD characteristics leads to problems with the very definition of ASD. Subjects that have ADOS summary scores that put them near the borderline of “mild ASD” versus “not ASD, but exhibiting ASD-like characteristics” requires the EEG classifier to make decisions that result in for which even human clinicians have low inter-rater agreement. This is exactly what we see in Table 5.b. The age trend is interesting as well: at 3 and 6 months, even the HRA− subjects are easily classified “correctly” as not-ASD. This may simply be because ASD brain function has not yet fully emerged. After 9 months, the HRA− subjects are more difficult to classify, perhaps because ASD-like brain function, associated with CSS summary scores that approach the borderline, is emerging.

This situation is remedied in Table 5.c where we allow 3 classification outcomes: ASD, not-ASD, and ‘uncertain’. The latter category occurs when the EEG features lead to a score that puts the subject very near the classification plane, as illustrated in Fig. 2. Using this approach, 116 subjects with data at 6 and 9 months were classified perfectly, with only 7 of the 116 labeled as “uncertain”. It may be that a longitudinal risk assessment model that updates with each new measurement might enable these uncertainties to be resolved as the child passes 12 months, or 18 months. This remains an area for future research.

Examining the results in Table 5.c, it appears that the lowest predictive accuracy occurs at 12 months. Also, the largest number of infants labeled as “uncertain” occurs at 9, 12, and 18 months. This seems to correlate with the shift in EEG feature values that occurs at around 12 months. By 36 months, the number of uncertain labels has decreased and the predictive accuracy has improved again. However, the predictive accuracy appears to be somewhat lower at 36 months. In Figs 8–12 it is quite apparent that the confidence intervals become much wider at 36 months, suggesting that all groups are becoming more diverse in their electrophysiological activity.

The greatest remaining challenge in the evaluation of nonlinear signal analysis methods is to discover the neural and behavioral meaning of the various EEG measures. Terms used to label the features used in this study are derived from physical systems. Entropy, determinism, laminarity, and so on have meaning in the context of turbulent fluid flow, for example. The RQA-derived values appeared to be similar, and distinctly different from SampE and DFA. Thus, regional plots (Figs 8–12) examined only SampE and DET. Further research is required to determine if more information, perhaps subtle, can be found in the array of RQA, or other nonlinear, signal features. Translating these mathematical constructs to networks of millions of neural generators cannot be done through analytical mathematics alone, but may require many empirical studies whereby differences in entropy, for example, are compared to many cognitive and behavioral phenotypes. Machine learning algorithms are useful for recognizing that significant correlations between patterns of features and outcomes exist, but these are unable to interpret these correlations in electrophysiological terms. One of the goals of this study is to encourage the broader neuroscience community to become involved in this research and contribute to a better and deeper understanding of the relationship between nonlinear (and multiscale) analysis of electrophysiological signals and their meaning in the context of brain-behavior relationships.

## Conclusions

The results presented in this paper are consistent with and greatly extend our previous study with a subset from this cohort of infants (Bosl *et al*., 2011). Nonlinear analysis of EEG signals extracts information that is significantly different in children who develop ASD, as early as 3 months of age. Predictions of the diagnostic outcomes were highly accurate using measurements as early as 3 months of age. Our analytic approach was not only associated with the binary outcome, but also strongly correlated with symptom severity as measured by CSS scores. Developmental trajectories of SampE and DET in key brain regions associated with ASD revealed significant differences between the three groups. In general, the ASD group diverged from the LRC− group at early ages in the left temporal and right temporal-parietal regions, and diverged later, after 18 months, in frontal regions. Predicted severity scores were significantly correlated with actual scores using EEG measurements taken at 3 months of age and older. This suggests that EEG measurement using the method presented here is a promising technology for monitoring neural development in a broad population of children. Future research with larger and more diverse populations is needed to determine the clinical applicability of this approach to ASD detection in general populations. As our results when combining 6- and 9-month suggest, longitudinal studies that create trajectories rather than point measurements at a single age might yield further insights.
